# Order of resolution and recurrence of inflammatory symptoms in lipedema: a longitudinal study of 1,300 patients

**DOI:** 10.1590/1677-5449.202600562

**Published:** 2026-09-07

**Authors:** Alexandre Campos Moraes Amato, Juliana Lelis Spirandeli Amato, Daniel Augusto Benitti

**Affiliations:** 1 Amato Duo Instituto, São Paulo, SP, Brasil.; 2 Valens Medical Center, Campinas, SP, Brasil.

**Keywords:** lipedema, inflammatory symptoms, resolution order, bruising, patient-reported outcomes, longitudinal study, lipedema, sintomas inflamatórios, ordem de resolução, equimose, desfechos relatados pelo paciente, estudo longitudinal

## Abstract

**Background:**

The Questionário de avaliação sintomática do lipedema (QuASiL – Lipedema Symptom Assessment Questionnaire) assesses 13 distinct inflammatory symptoms of lipedema, but whether these symptoms resolve and recur simultaneously or follow a consistent order remains unknown.

**Objectives:**

To characterize the persistence hierarchy of the 13 QuASiL inflammatory symptoms in a retrospective lipedema cohort under heterogeneous conservative treatment, using 6 complementary rank-order analyses and testing the null hypothesis that symptoms resolve with equal magnitude.

**Methods:**

This retrospective longitudinal study included 1,300 patients who were clinically diagnosed with lipedema by a vascular surgeon and who completed the QuASiL on at least 2 occasions (minimum 14-day interval; median follow-up 181 days, IQR 92–380, maximum 1,787 days). A consensus ranking was computed across all analyses. A sensitivity analysis was conducted using the conventional *T*_0_ = first visit, *T*_1_ = last visit convention, and a Kaplan-Meier survival analysis estimated the time (in absolute days) to ≥ 50% reduction from baseline per symptom.

**Results:**

The symptoms did not resolve simultaneously (Friedman χ^2^ (12) = 168.08, p < 0.001). All 13 symptoms improved during follow-up, showing a consistent order across the 6 analyses. A slower-resolving core was identified: bruising (consensus rank 1.7), swelling (2.0), touch sensitivity (4.3), leg heaviness (4.7), and fatigue (4.7). A rapidly-responding group comprised skin irritation (66.4% proportional reduction), itching (62.0%), and walking difficulty (62.6%). Even the most resistant symptom (bruising) achieved a mean 39.1% proportional reduction, with half of patients reaching ≥ 50% improvement. During worsening, swelling and pressure/tension re-emerged first. The sensitivity analysis (T0 = first, T1 = last) produced an essentially identical ranking (Spearman ρ = 0.945, p < 10^−5^). The Kaplan-Meier analysis estimated a median of 546 days for bruising vs 207 days for itching to reach ≥ 50% reduction from baseline (log-rank χ2 = 44.6, p < 10^−10^); the fastest-resolving symptom was heat/burning at 203 days.

**Conclusions:**

In this retrospective cohort of patients under heterogeneous conservative treatment, the 13 QuASiL inflammatory symptoms improved following a consistent order across 6 independent analyses, with bruising and swelling as the most persistent symptoms.

## Resumo

**Contexto:**

O Questionário de avaliação sintomática do lipedema (QuASiL) avalia 13 sintomas inflamatórios distintos do lipedema, porém desconhece-se se esses sintomas se resolvem e recidivam simultaneamente ou se seguem uma ordem consistente.

**Objetivos:**

Caracterizar a hierarquia de persistência dos 13 sintomas inflamatórios do QuASiL em uma coorte retrospectiva de pacientes com lipedema submetidos a tratamento conservador heterogêneo, utilizando seis análises complementares de ordenação e testando a hipótese nula de que os sintomas são resolvidos com a mesma magnitude.

**Métodos:**

Este estudo longitudinal retrospectivo foi desenvolvido com 1.300 pacientes com diagnóstico clínico de lipedema realizado por cirurgião vascular e que completaram o QuASiL em pelo menos duas ocasiões (intervalo mínimo de 14 dias; mediana de acompanhamento de 181 dias, intervalo interquartil de 92–380, máximo 1.787 dias). Uma classificação de consenso foi calculada a partir de todas as análises. Realizou-se uma análise de sensibilidade com a definição convencional *T*_0_ = primeira visita, *T*_1_ = última visita, e uma análise de sobrevida de Kaplan-Meier estimou o tempo (em dias absolutos) para uma redução ≥ 50% em relação ao valor inicial para cada sintoma.

**Resultados:**

Os sintomas não cessaram simultaneamente (teste de Friedman χ^2^ (12) = 168,08, p < 0,001). Todos os 13 sintomas apresentaram melhora durante o acompanhamento, exibindo uma ordem consistente nas seis análises. Identificou-se o grupo de resolução mais lenta: equimose (classificação de consenso 1,7), edema (2,0), sensibilidade ao toque (4,3), sensação de peso nas pernas (4,7) e fadiga (4,7). O outro grupo apresentou resposta rápida, irritação da pele (redução proporcional de 66,4%), prurido (62,0%) e dificuldade para caminhar (62,6%). Mesmo o sintoma mais resistente (equimose) apresentou redução proporcional média de 39,1%, com metade dos pacientes atingindo uma melhora ≥ 50%. Durante episódios de piora, o edema e a sensação de pressão/tensão reapareceram primeiro. A análise de sensibilidade (*T*_0_ = primeira visita, *T*_1_ = última visita) produziu uma classificação essencialmente idêntica (ρ de Spearman = 0,945; p < 10^−5^). A análise de Kaplan-Meier estimou uma mediana de 546 dias para a equimose e de 207 dias para o prurido até se atingir uma redução ≥ 50% em relação ao valor inicial (log-rank χ^2^ = 44,6; p < 10^−10^); o sintoma com resolução mais rápida foi a sensação de calor/queimação, com 203 dias.

**Conclusões:**

Nesta coorte retrospectiva de pacientes submetidos a tratamento conservador heterogêneo, os 13 sintomas da escala QuASiL apresentaram melhora seguindo uma ordem consistente nas seis análises independentes, sendo a equimose e o edema os sintomas mais persistentes.

## INTRODUCTION

Lipedema is a chronic, progressive disorder of adipose tissue characterized by bilateral, symmetric, and disproportionate fat deposition primarily affecting the lower extremities.^1,2^ Estimated to affect 7% to 12% of women, it remains frequently underdiagnosed and misdiagnosed as simple obesity or lymphedema.^2-4,5^ Beyond the morphological changes, patients typically present with a constellation of inflammatory and sensory symptoms including pain, easy bruising, leg heaviness, swelling, and tenderness, which profoundly affect quality of life.^6-8^

The pathophysiology of lipedema involves chronic low-grade inflammation with macrophage infiltration, dilated blood microvessels, angiogenesis, and increased interstitial fluid.^9,10^ Studies have reported altered inflammatory signaling pathways in lipedema adipocytes, with M2-polarized macrophage predominance indicating chronic low-grade inflammation and tissue remodeling.^11,12^ These inflammatory processes manifest clinically as the diverse symptom profile that defines the condition.

Based on Rapprich, Dingler & Podda’s Questionnaire for measuring disorders of lipedema by visual analogue scales,^13^ the Questionário de avaliação sintomática do lipedema (QuASiL – Lipedema Symptom Assessment Questionnaire) was developed and validated to systematically evaluate this multidimensional symptom burden, which comprises 13 inflammatory/sensory items spanning pain/sensory, vascular, inflammatory, skin, and functional domains.^14,15^ While this instrument and others have been used to assess overall treatment response,^16-18^ no study has investigated whether individual symptoms follow a consistent order of resolution during treatment or recurrence during relapse.

Understanding the temporal sequence of symptom resolution has important clinical implications. Conservative treatment of lipedema — including compression therapy, manual lymphatic drainage, and exercise — produces measurable reductions in overall symptom burden,^19,20^ but the sequence in which individual symptoms respond has not been characterized. Bruising is one of the hallmark clinical features and a key diagnostic criterion of lipedema, reflecting the characteristic capillary fragility of the condition.^1,21,22^ Szolnoky et al. demonstrated that capillary fragility measurement can differentiate lipedema from obesity, suggesting distinct vascular vulnerability in affected tissue.^23^ Whether this vascular fragility translates into a differential treatment response compared to other symptoms has not been investigated.

In this retrospective observational study, we aimed: (1) to characterize the persistence hierarchy of the 13 QuASiL inflammatory symptoms by integrating 6 complementary rank-order analyses, testing the null hypothesis that symptoms resolve with equal magnitude; (2) to identify which symptoms involve the greatest residual burden during follow-up and which have the largest proportional reduction; (3) to determine, in a subgroup of patients with ≥ 4 visits and a documented worsening phase, which symptoms cross a pre-specified clinical threshold earliest during relapse; and (4) to test the *a priori* hypothesis that bruising, as a marker of capillary fragility, is the most persistent symptom. Because the study is observational and treatment was not standardized, the findings are interpreted as exploratory and hypothesis-generating rather than prognostic.

## METHODS

### Study design and population

This retrospective longitudinal observational study analyzed data from patients with lipedema who completed the QuASiL at a specialist vascular center. The study was reported following STROBE guidelines.^24^ Ethics approval was obtained from the institutional research ethics committee (CAAE: 94620125.0.0000.5450; Opinion 8.163.019). All procedures were in accordance with the ethical standards of the institutional research committee and the 1964 Helsinki Declaration and its later amendments. The informed consent requirement was waived by the ethics committee due to the study’s retrospective nature and its use of anonymized data.

All patients were evaluated at a single tertiary vascular surgery center. Lipedema was clinically diagnosed by one of the authors, a vascular surgeon, during a medical consultation and using the Brazilian Consensus Statement criteria,^5^ prior to administering the QuASiL. All patients subsequently received the conservative treatment routinely offered at the service, including graded compression therapy, manual lymphatic drainage, supervised exercise, and dietary guidance in heterogeneous regimens reflecting individual clinical judgment and patient adherence, consistent with the retrospective nature of the study. Specific treatment protocols, doses, and adherence were not centrally recorded in the electronic database. Lipedema staging was not systematically captured in the database and could not be analyzed as a modifier, which we acknowledge as a limitation.

Inclusion criteria were: (1) clinical diagnosis of lipedema established by a vascular surgeon, with a valid email address for identification; (2) at least 2 QuASiL assessments; and (3) minimum 14-day follow-up interval between the first and last assessments. Patients with a single assessment, follow-up < 14 days, or who underwent surgical intervention (liposuction) during the observation window were excluded.

The follow-up interval varied substantially across patients, as expected for a retrospective cohort. For the entire longitudinal cohort (N = 1,300), the median interval between the first and last QuASiL assessment was 181 days (IQR 92–380; range 14–1,787; ~6 months median, with a maximum of ~4.9 years). Subgroup-level follow-up distributions are reported in Supplementary Table S1. The median number of visits per patient was 2 (IQR 2–4; range 2–18), with 25.5% of patients having ≥ 4 visits.

### Data source

The QuASiL is a 15-item self-report questionnaire developed and validated at the authors’ institution, scored on a rating scale of 0–10. It includes 13 inflammatory/sensory items (Q1–Q13: pain, touch sensitivity, bruising tendency, pressure/tension, heat/burning, cold sensation, cramps, leg heaviness, fatigue, swelling, skin irritation, itching, walking difficulty), 1 quality-of-life item (Q14), and 1 aesthetic dissatisfaction item (Q15).^14^ The inflammatory score (sum of Q1–Q13) ranges from 0 to 130, with higher scores indicating worse symptoms. Only the 13 individual inflammatory symptoms were considered in the present analysis.

Items Q14 (global quality of life) and Q15 (aesthetic dissatisfaction) were not analyzed here because they represent global subjective outcomes rather than discrete inflammatory/sensory symptoms; the temporal behavior of these composite constructs is conceptually distinct from that of the individual symptoms and does not bear on the hierarchy question addressed in this study. Q14 and Q15 are the focus of a separate companion study by our group.

These 13 symptoms were grouped into 5 exploratory pathophysiological subdomains: pain/sensory (Q1: pain, Q2: touch sensitivity), vascular (Q3: bruising, Q6: cold sensation, Q7: cramps), inflammatory (Q4: pressure/tension, Q5: heat/burning, Q8: leg heaviness, Q10: swelling), skin (Q11: skin irritation, Q12: itching), and functional (Q9: fatigue, Q13: walking difficulty).

### Longitudinal data preparation

For each patient with multiple assessments, the data were organized into baseline (*T*_0_) and follow-up (*T*_1_) timepoints. For individual symptom scores, *T*_0_ was defined as the maximum score from the first half of the visits (worst initial state) and *T*_1_ as the minimum score from the second half (best achieved state after treatment). This strategy captures the maximum possible improvement for each patient. Change scores were calculated as *T*_0_ minus *T*_1_, with positive values indicating improvement.

We acknowledge that this worst-to-best definition biases the *Δ* toward positive values by construction and may overestimate the absolute magnitude of improvement. However, because all analyses contrast symptoms relative to each other within the same patient, this bias is shared across symptoms and does not distort the ranking. Nevertheless, to demonstrate that the persistence hierarchy does not depend on this choice, we repeated the 5 analyses that use the *T*_0_/*T*_1_ framing (Analyses 1, 2, 3, 5 and 6) with the conventional definition *T*_0_ = first available assessment, *T*_1_ = last available assessment (Sensitivity analysis, below). Analysis 4 (emergence) operated on raw multi-visit trajectories and did not depend on the *T*_0_/*T*_1_ definition.

### Sensitivity analysis (T_0_ = first visit, T_1_ = last visit)

The 5 *T*_0_/*T*_1_ analyses were recomputed using the conventional first-to-last definition. A Spearman rank correlation compared the sensitivity consensus ranking to the primary (worst-best) consensus ranking. The Friedman test was re-applied to patients with all 13 symptoms active on the first visit.

### Survival analysis (time-to-event in absolute days)

To address the limitation of normalized (0–1) time metrics used in Analyses 3 and 4 and to provide a measure independent of the *T*_0_/*T*_1_ convention, a Kaplan-Meier analysis was performed for patients with ≥ 4 visits and baseline symptom scores ≥ 3. Two clinically meaningful events were defined per symptom: (A) first visit at which the score dropped to ≤ 50% of baseline, and (B) first visit at which the score dropped to ≤ 2 (mild/absent). Patients who did not reach the threshold were censored at their last assessment. Survival functions were estimated with Greenwood 95% CIs; median times in days were extracted. Pairwise log-rank tests compared selected extreme symptoms (bruising vs itching, bruising vs skin irritation, swelling vs walking difficulty).

### Statistical analysis

Six complementary analytical approaches were employed to determine the order of symptom resolution and emergence:

**Analysis 1 — Proportional reduction.** Among patients with meaningful overall inflammatory improvement (composite score decrease > 10 points), the proportional reduction of each symptom was calculated per patient as (T_0_ - T_1_)/T_0_ x 100 for each patient i who had that symptom at baseline (score ≥ 3), and then averaged across patients. Symptoms with a lower proportional reduction were considered more resistant to treatment. The complete resolution rate (*T*_1_ = 0) was also calculated.
**Analysis 2 — Residual burden.** Among strong responders (top 25th percentile of inflammatory improvement), mean *T*_1_ scores and the proportion of patients still scoring ≥ 3 (active symptom) and ≥ 5 (high symptom) at follow-up were calculated for each symptom.
**Analysis 3 — Time-to-50% resolution.** For patients with ≥ 4 visits who improved (composite decrease > 10), symptom values were normalized to baseline (fraction of initial score) and time was normalized to 0–1 (first to last visit). The timepoint at which each symptom first dropped below 50% of its baseline was identified. Higher values indicate slower resolution.
**Analysis 4 — Symptom emergence order.** For patients with ≥ 4 visits who experienced a worsening phase (defined as a period of low symptoms followed by an increase of > 10 composite points), the timepoint at which each symptom first exceeded a score of 4 (from a controlled state of ≤ 2) was recorded. Earlier emergence indicates that the symptom appeared first during relapse.
**Analysis 5 — Pairwise persistence dominance.** For each pair of symptoms among patients who had both symptoms at baseline (score ≥ 3 each) and who improved overall, the proportion of cases in which symptom A had a higher *T*_1_ score than symptom B was calculated. A mean dominance score was computed for each symptom across all 12 pairings.
**Analysis 6 — Resolution order concordance.** For patients in whom all 13 symptoms were active at baseline (score ≥ 3 each), proportional reduction values were ranked within each patient (rank 1 = least reduction, rank 13 = most reduction). The Friedman test was used to determine whether the ranking was consistent across patients. Mean ranks were computed for each symptom.

Consensus ranking: each analysis yielded a ranking of the 13 symptoms from most persistent (rank 1) to least persistent (rank 13). The final consensus ranking was computed as the mean rank across all 6 analyses. To assess the robustness of this ranking, a bootstrap procedure (10,000 iterations) was performed by resampling the 6 analyses with replacement and recomputing the mean rank (a rank-aggregation/Borda-type approach^25,26^). The 95% bootstrap CIs for each symptom’s consensus rank were derived from the 2.5th and 97.5th percentiles of the bootstrap distribution. Non-overlapping CIs between adjacent ranked symptoms were used to identify statistically supported tier boundaries.

All analyses were performed in Python 3.12 with the Pandas, NumPy, SciPy, and Matplotlib libraries. Statistical significance was set at p < 0.05 (2-tailed).

**Methodological references.** The Friedman test for concordance of paired rankings followed Friedman 1937.^27^ The pairwise dominance approach is a rank-based non-parametric comparison, as discussed in Conover 1999.^25^ Rank aggregation (mean rank/Borda count) followed Kendall 1975.^26^ Bootstrap confidence intervals followed Efron & Tibshirani 1993.^28^ The Kaplan-Meier product-limit estimator^29^ and the log-rank test^30^ were implemented from first principles using NumPy and SciPy.

## RESULTS

### Study population

Of 6,037 QuASiL records representing 3,206 patients, 1,856 were excluded for having only a single visit and 50 were excluded for insufficient follow-up, yielding 1,300 patients for analysis. Of these, 660 (50.8%) had meaningful inflammatory improvement (composite decrease > 10 points) and were used in resolution analyses. A total of 335 patients (25.8%) were strong responders (top 25th percentile, composite decrease ≥ 30 points). For trajectory analyses, 331 patients had ≥ 4 visits, of whom 196 were improvers.

### The resolution order is non-random

The Friedman test, applied to 117 patients in whom all 13 symptoms were active at baseline (score ≥ 3), confirmed that the resolution order across symptoms was significantly non-random (*χ*^2^ [12] = 168.08, p < 0.001). This indicates that, in this retrospective cohort, inflammatory symptoms did not resolve simultaneously and followed a consistent order across the analyses performed — a pattern whose prognostic value requires prospective confirmation.

### Proportional reduction analysis

Among 660 improvers, proportional reduction varied widely across symptoms (Table 1). Bruising had the lowest mean proportional reduction (39.1%) and the lowest complete resolution rate (9.3%). Skin irritation had the highest proportional reduction (66.4%) and a high complete resolution rate (41.9%). The difference between the most resistant symptom (bruising, 39.1%) and the most responsive symptom (skin irritation, 66.4%) was 27.3 percentage points, a 1.7-fold difference in treatment responsiveness.

**Table 1:** Proportional reduction of individual symptoms among patients with inflammatory improvement (N = 660). Analysis population: patients with meaningful overall inflammatory improvement (composite score decrease > 10 points), N = 660 (50.8% of the longitudinal cohort). For each symptom, only patients with baseline score ≥ 3 on that symptom were included in the per-symptom statistics.

**Rank**	**Symptom (QuASiL item)**	**Subdomain**	**Mean *T*_0_**	**Mean *T*_1_**	**Proportional reduction (%)**	**Complete resolution (%)**
1	Q3: Bruising	Vascular	7.9	4.7	39.1	9.3
2	Q10: Swelling	Inflammatory	8.8	5.1	41.7	6.5
3	Q2: Touch sensitivity	Pain/Sensory	7.6	4.1	45.3	13.8
4	Q1: Pain	Pain/Sensory	7.4	3.9	46.2	11.3
5	Q9: Fatigue	Functional	8.5	4.6	46.8	10.0
6	Q8: Leg heaviness	Inflammatory	8.5	4.5	47.1	12.1
7	Q4: Pressure/tension	Inflammatory	7.9	4.0	48.9	13.1
8	Q7: Cramps	Vascular	6.3	2.7	58.2	28.3
9	Q6: Cold sensation	Vascular	6.6	2.8	58.8	37.8
10	Q12: Itching	Skin	6.4	2.5	62.0	32.8
11	Q13: Walking difficulty	Functional	6.5	2.5	62.6	43.7
12	Q5: Heat/burning	Inflammatory	7.1	2.6	64.4	35.2
13	Q11: Skin irritation	Skin	6.6	2.3	66.4	41.9

Ranked by proportional reduction (ascending); rank 1 = most resistant to treatment. Proportional reduction calculated per patient as (T_0_ − T_1_)/ T_0_ ×100 and averaged across patients. Only patients with baseline scores ≥ 3 for each symptom were included. T_0_ = baseline (worst state); T_1_ = follow-up (best achieved state).

### Residual burden after treatment

Among the 335 strong responders (Table 2), swelling and bruising had the highest residual scores at *T*_1_ (both 3.9/10), with 67.2% and 62.7% of patients still scoring ≥ 3, respectively. In contrast, walking difficulty had the lowest residual burden (mean *T*_1_ = 1.1), with only 18.2% still scoring ≥ 3. Notably, bruising also had the highest residual score among strong responders (40.9% still ≥ 5), consistent with its structural vascular basis — which responds more gradually than mediator-driven symptoms, although this does not indicate an absence of treatment effect.

**Table 2:** Residual symptom burden among strong responders (N = 335). Analysis population: strong responders, defined as top 25th percentile of inflammatory composite improvement (*Δ* ≥ 30 points), N = 335 (25.8% of the longitudinal cohort). All 335 patients contribute to each symptom row.

**Rank**	**Symptom**	**Subdomain**	**Mean *T*_0_**	**Mean *T*_1_**	**Still active ≥ 3 (%)**	**Still high ≥ 5 (%)**
1	Q10: Swelling	Inflammatory	9.0	3.9	67.2	40.0
2	Q3: Bruising	Vascular	7.8	3.9	62.7	40.9
3	Q9: Fatigue	Functional	8.8	3.3	59.7	32.8
4	Q8: Leg heaviness	Inflammatory	8.6	3.3	58.2	30.1
5	Q2: Touch sensitivity	Pain/Sensory	7.5	3.2	58.2	30.4
6	Q4: Pressure/tension	Inflammatory	8.2	3.0	54.0	26.3
7	Q1: Pain	Pain/Sensory	7.4	3.0	53.7	26.0
8	Q6: Cold sensation	Vascular	4.6	1.6	27.8	14.6
9	Q5: Heat/burning	Inflammatory	6.0	1.5	24.8	11.6
10	Q7: Cramps	Vascular	4.9	1.4	20.9	9.9
11	Q12: Itching	Skin	4.5	1.2	21.5	9.0
12	Q11: Skin irritation	Skin	5.0	1.2	22.1	9.3
13	Q13: Walking difficulty	Functional	4.0	1.1	18.2	11.0

Ranked by mean T_1_ score (descending); ties resolved by proportion still active (≥ 3), descending. Strong responders defined as top 25th percentile of inflammatory composite improvement (Δ ≥ 30 points).

### Time-to-50% resolution

Among 196 improvers with ≥ 4 visits (Table 3), bruising had the slowest time-to-50% resolution (mean normalized time = 0.727, median = 0.899): half of the patients achieved ≥ 50% improvement, though it required more of the follow-up period than any other symptom. In contrast, itching was the fastest to reach 50% resolution (mean = 0.525), with 80.9% of patients achieving this threshold. The trajectory analysis revealed that bruising consistently remained the highest relative to baseline across all timepoints (Figure 1).

**Table 3:** Time-to-50% resolution among patients with ≥ 4 visits (N = 196). Analysis population: patients with ≥ 4 QuASiL assessments AND overall improvement (*Δ* inflammatory > 10), N = 196 (15.1% of the longitudinal cohort). For each symptom, only patients with baseline score ≥ 3 on that symptom contribute to the per-symptom N.

**Rank**	**Symptom**	**Subdomain**	**N**	**Mean normalized time**	**Median normalized time**	**Never reached 50% (%)**
1	Q3: Bruising	Vascular	172	0.727	0.899	49.4
2	Q2: Touch sensitivity	Pain/Sensory	176	0.667	0.667	42.6
3	Q10: Swelling	Inflammatory	192	0.650	0.667	40.1
4	Q1: Pain	Pain/Sensory	181	0.628	0.667	37.0
5	Q9: Fatigue	Functional	191	0.622	0.625	36.1
6	Q8: Leg heaviness	Inflammatory	184	0.619	0.600	36.4
7	Q4: Pressure/tension	Inflammatory	184	0.598	0.571	27.7
8	Q6: Cold sensation	Vascular	95	0.584	0.400	36.8
9	Q7: Cramps	Vascular	124	0.568	0.429	31.5
10	Q11: Skin irritation	Skin	107	0.555	0.500	26.2
11	Q13: Walking difficulty	Functional	99	0.533	0.400	22.2
12	Q5: Heat/burning	Inflammatory	132	0.531	0.400	22.0
13	Q12: Itching	Skin	94	0.525	0.483	19.1

Ranked by mean normalized time (descending); rank 1 = slowest to resolve. Normalized time: 0 = first visit, 1 = last visit. Only patients with baseline scores ≥ 3 for each symptom were included.

**Figure 1:**
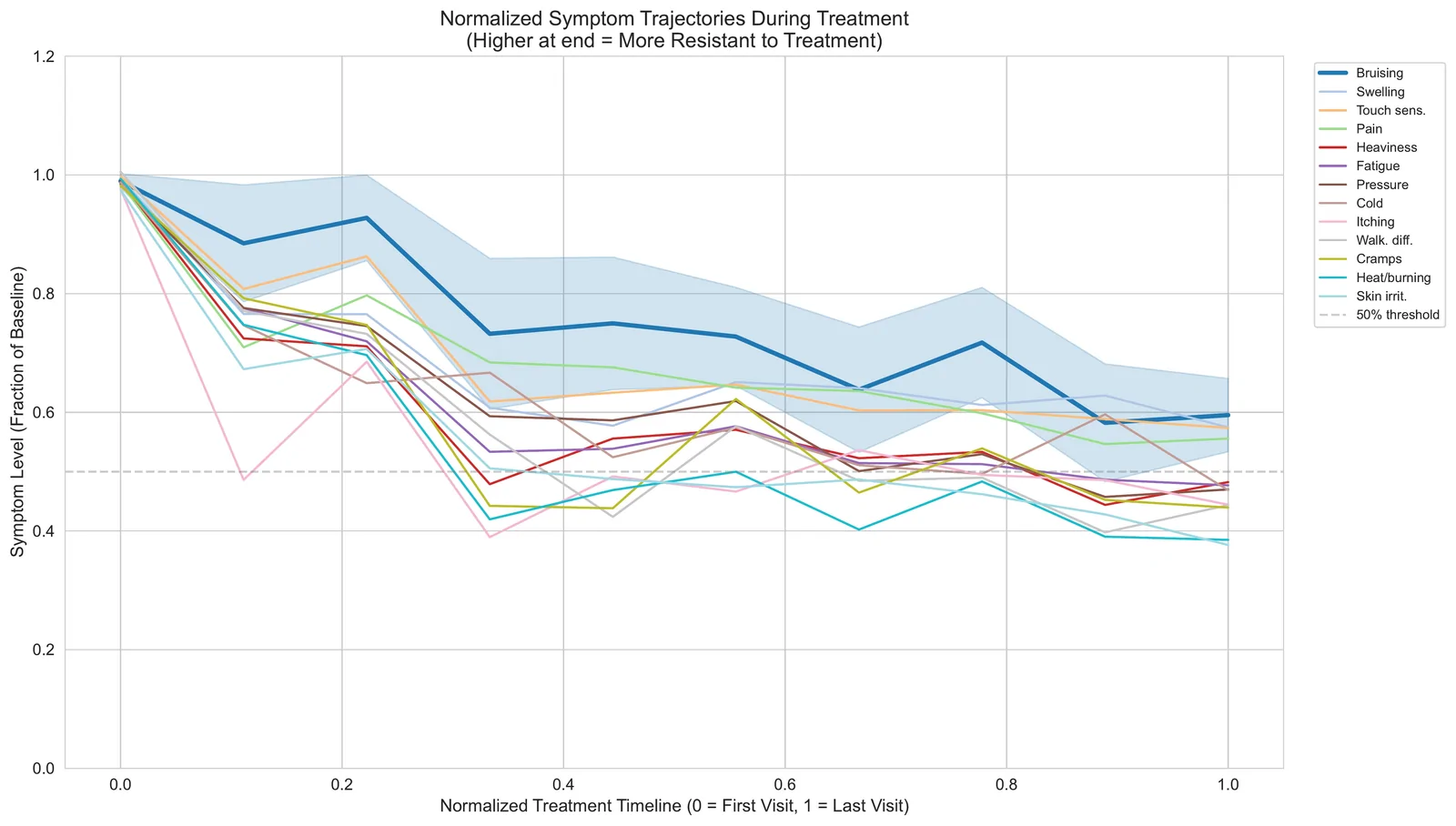
Normalized symptom trajectories during treatment in patients with ≥ 5 visits (N = 125). Each line represents the mean trajectory of 1 symptom, normalized to baseline (1.0 = baseline level). Bruising (bold line) consistently showed the highest residual level across the entire treatment period. The dashed horizontal line indicates the 50% resolution threshold.

### Symptom emergence order during worsening

Among 145 patients with an identifiable worsening phase (Table 4), swelling was the first symptom to re-emerge (mean normalized time = 0.550, with 81.6% of eligible patients experiencing re-emergence), followed closely by pressure/tension (0.554, 72.7%) and bruising (0.606, 68.3%). Walking difficulty (0.846, 27.0%) and itching (0.843, 29.0%) were the last to emerge during relapse (Figure 2).

**Table 4:** Symptom emergence order during worsening phases (N = 145). Analysis population: patients with ≥ 4 QuASiL assessments who experienced a worsening phase (score increase >10 composite points after a nadir), N = 145 (11.2% of the longitudinal cohort). *N = number of patients with each symptom controlled (score ≤ 2) at the worsening nadir, being thus eligible for the emergence analysis of that specific symptom.

**Rank**	**Symptom**	**Subdomain**	N*	**Mean emergence time**	**Patients with emergence (%)**
1	Q10: Swelling	Inflammatory	38	0.550	81.6
2	Q4: Pressure/tension	Inflammatory	77	0.554	72.7
3	Q3: Bruising	Vascular	60	0.606	68.3
4	Q1: Pain	Pain/Sensory	57	0.623	66.7
5	Q8: Leg heaviness	Inflammatory	61	0.633	65.6
6	Q9: Fatigue	Functional	54	0.676	61.1
7	Q5: Heat/burning	Inflammatory	116	0.722	48.3
8	Q11: Skin irritation	Skin	110	0.740	45.5
9	Q2: Touch sensitivity	Pain/Sensory	58	0.745	46.6
10	Q6: Cold sensation	Vascular	106	0.788	37.7
11	Q7: Cramps	Vascular	101	0.812	33.7
12	Q12: Itching	Skin	107	0.843	29.0
13	Q13: Walking difficulty	Functional	111	0.846	27.0

Ranked by mean emergence time (ascending); rank 1 = first to appear during relapse. Emergence defined as score reaching ≥ 4 from a controlled state (≤ 2).

* N = number of patients with each symptom controlled (score ≤ 2) at the worsening phase nadir, being thus eligible for the emergence analysis of that specific symptom; percentages reflect the proportion of N who experienced re-emergence, not the proportion of the overall 145 patients.

**Figure 2:**
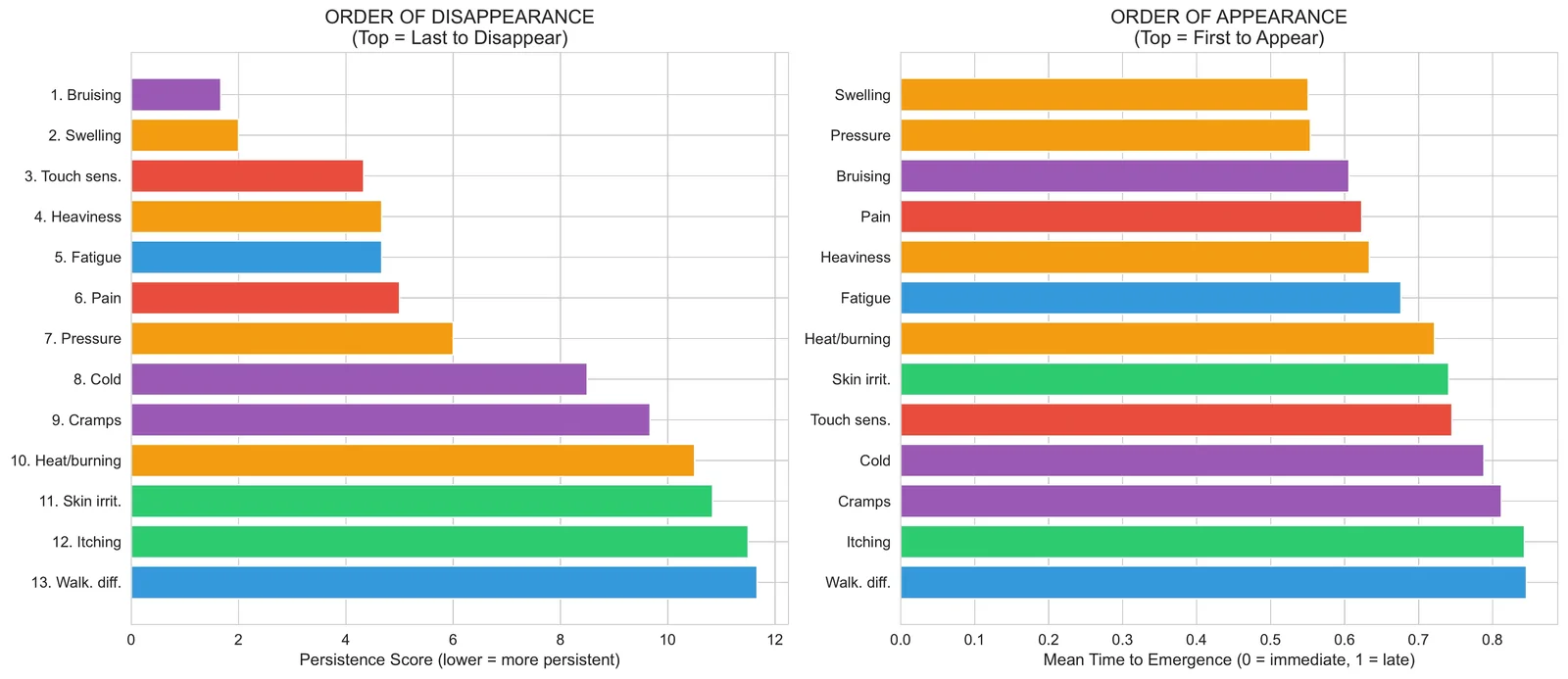
Comparison of the disappearance order (left panel) and appearance order (right panel). The left panel shows the consensus persistence ranking (lower = more persistent). The right panel shows the emergence order during worsening phases (lower = first to appear). Colors indicate pathophysiological subdomain (vascular: purple; inflammatory: orange; pain/sensory: red; functional: blue; skin: green).

### Pairwise persistence dominance

The pairwise analysis confirmed the persistence hierarchy (Table 5). Swelling had the highest overall dominance score (0.635), meaning it outlasted the other symptom in 63.5% of head-to-head comparisons. Bruising ranked second (0.568). Walking difficulty had the lowest dominance score (0.220). The pairwise dominance heatmap (Figure 3) demonstrated a clear block structure, with a persistent core group (swelling, bruising, fatigue, heaviness) consistently outlasting a responsive group (skin irritation, itching, walking difficulty, heat/burning).

**Table 5:** Pairwise persistence dominance scores.

**Rank**	**Symptom**	**Subdomain**	**Dominance score**
1	Q10: Swelling	Inflammatory	0.635
2	Q3: Bruising	Vascular	0.568
3	Q9: Fatigue	Functional	0.540
4	Q8: Leg heaviness	Inflammatory	0.521
5	Q2: Touch sensitivity	Pain/Sensory	0.484
6	Q4: Pressure/tension	Inflammatory	0.462
7	Q1: Pain	Pain/Sensory	0.451
8	Q6: Cold sensation	Vascular	0.297
9	Q7: Cramps	Vascular	0.270
10	Q5: Heat/burning	Inflammatory	0.236
11	Q12: Itching	Skin	0.232
12	Q11: Skin irritation	Skin	0.224
13	Q13: Walking difficulty	Functional	0.220

Dominance score = mean proportion of pairwise comparisons in which the symptom outlasts the other symptom at T_1_ (scores > 0.5 indicate that the symptom is more persistent than average).

**Figure 3:**
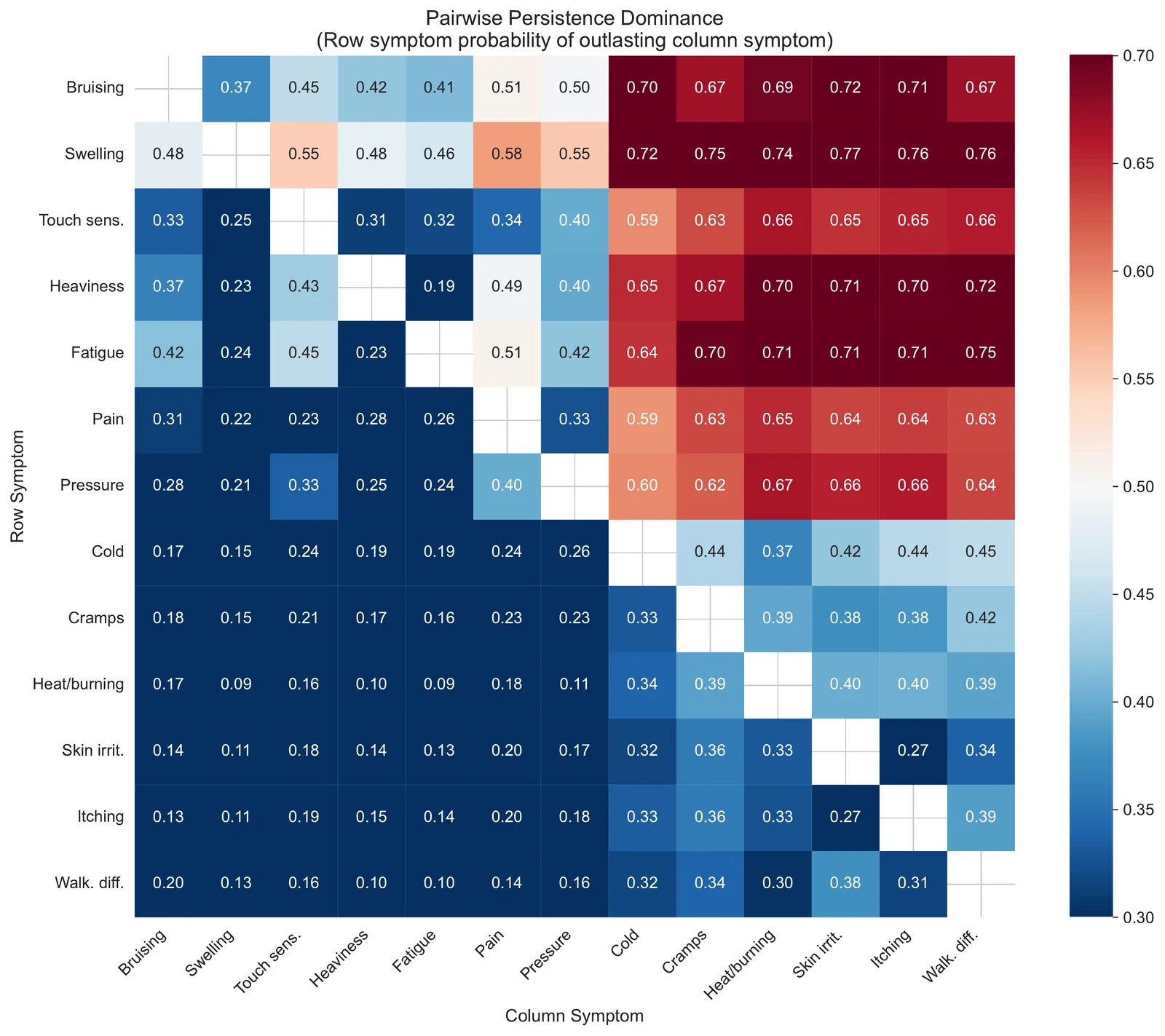
Pairwise persistence dominance heatmap. Each cell shows the probability that the row symptom would outlast the column symptom at follow-up. Values > 0.5 (red) indicate that the row symptom is more persistent. A clear block structure separates the persistent core symptom (upper-left) from the responsive periphery symptom (lower-right).

### Consensus ranking

The consensus ranking across all 6 analyses (Table 6, Figure 4) identified a clear hierarchy. Two distinct symptom groups emerged:

**Table 6:** Consensus ranking of symptom persistence across 6 analyses.

**Consensus rank**	**Symptom**	**Subdomain**	**Mean rank**	**95% CI**	**Group**
1	Q3: Bruising	Vascular	1.7	1.17–2.33	Persistent core
2	Q10: Swelling	Inflammatory	2.0	1.17–3.00	Persistent core
3	Q2: Touch sensitivity	Pain/Sensory	4.3	2.67–6.50	Persistent core
4	Q8: Leg heaviness	Inflammatory	4.7	3.83–5.50	Persistent core
5	Q9: Fatigue	Functional	4.7	3.67–5.67	Persistent core
6	Q1: Pain	Pain/Sensory	5.0	4.17–6.00	Persistent core
7	Q4: Pressure/tension	Inflammatory	6.0	4.33–7.00	Persistent core
**— TIER BOUNDARY (non-overlapping bootstrap CIs) —**					
8	Q6: Cold sensation	Vascular	8.5	8.00–9.17	Responsive periphery
9	Q7: Cramps	Vascular	9.7	8.83–10.50	Responsive periphery
10	Q5: Heat/burning	Inflammatory	10.5	8.67–12.00	Responsive periphery
11	Q11: Skin irritation	Skin	10.8	9.33–12.17	Responsive periphery
12	Q12: Itching	Skin	11.5	10.67–12.33	Responsive periphery
13	Q13: Walking difficulty	Functional	11.7	10.33–12.67	Responsive periphery

Consensus rank computed as the mean rank across all 6 analyses (proportional reduction, residual burden, time-to-50%, emergence order, pairwise dominance, and Friedman concordance). Lower mean rank = more persistent. The 95% CIs were derived from 10,000 bootstrap iterations (resampling of analyses). The tier boundary between ranks 7 and 8, the only non-overlapping gap in the ranking, represents the statistically supported separation between persistent core symptoms and responsive periphery symptoms.

**Figure 4:**
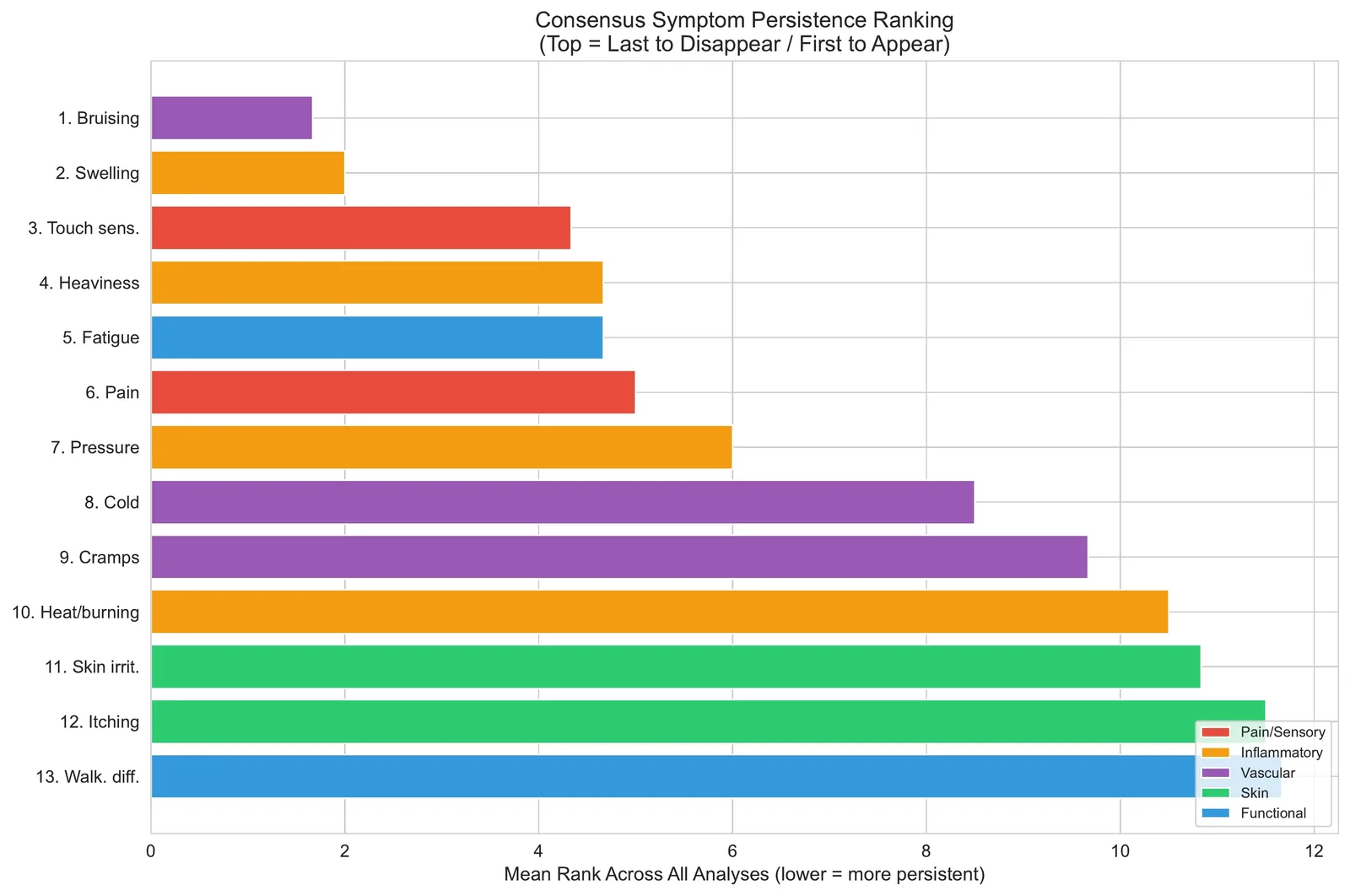
Consensus persistence ranking of the 13 QuASiL inflammatory symptoms. Bars represent the mean rank across 6 independent analyses. Colors indicate the pathophysiological subdomain. Bruising (rank 1.7) and swelling (rank 2.0) form the most persistent pair, while walking difficulty (rank 11.7) and itching (rank 11.5) form the most responsive pair.

**Persistent core** (consensus rank 1–7): bruising (1.7), swelling (2.0), touch sensitivity (4.3), leg heaviness (4.7), fatigue (4.7), pain (5.0), and pressure/tension (6.0). These symptoms showed mean proportional reductions of 39% to 49% and a high residual burden.
**Responsive periphery** (consensus rank 8–13): cold sensation (8.5), cramps (9.7), heat/burning (10.5), skin irritation (10.8), itching (11.5), and walking difficulty (11.7). These symptoms showed mean proportional reductions of 58% to 66% and a low residual burden.

Bootstrap analysis confirmed the statistical robustness of this 2-tier structure. The 95% CIs for pressure/tension (rank 7; 95% CI 4.33–7.00) and cold sensation (rank 8; 95% CI 8.00–9.17) did not overlap, identifying the boundary between the persistent core and responsive periphery as the only statistically supported tier gap in the ranking. Within each tier, adjacent symptoms showed overlapping CIs, indicating that individual positions within a tier should be interpreted as approximate rather than precise ordinal rankings.

Bruising ranked first in 3 of 6 individual analyses (proportional reduction, time-to-50%, concordance), second in 2 others (residual burden, pairwise dominance), and third in the emergence analysis, achieving the highest consensus persistence score.

### Sensitivity analysis: consistency under the conventional T_0_/T_1_ definition

To verify that the persistence hierarchy was not an artifact of the worst-to-best *T*_0_/*T*_1_ definition, the 5 *T*_0_/*T*_1_ analyses were recomputed using *T*_0_ = first visit and *T*_1_ = last visit (Supplementary Table S2, Supplementary Figure S1). Under this more conservative definition, 566 patients (vs 660 in the primary analysis) met the improver criterion. The consensus ranking derived from the sensitivity cohort was in very close agreement with the primary ranking: Spearman rank correlation ρ = 0.945 (p = 1.1 × 10^−6^), with a maximum rank shift of 3 positions (Supplementary Table S3). Bruising remained #1 and swelling #2 in both analyses. The Friedman test for the sensitivity cohort also confirmed non-simultaneous resolution (*χ*^2^ [12] = 63.65, p = 4.9 × 10^−9^). These results indicate that the observed hierarchy is robust to the *T*_0_/*T*_1_ definition.

### Survival analysis: absolute time to symptom resolution

To express resolution kinetics in clinically interpretable time units, Kaplan-Meier analyses were performed on the subgroup with ≥ 4 visits and a baseline score ≥ 3 (Supplementary Table S4, Supplementary Figure S2). The median time from baseline to ≥ 50% reduction varied substantially: 546 days for bruising (~18 months), 381 days for touch sensitivity, 368 days for swelling, decreasing to 203 days for heat/burning and 205 days for cold sensation — a 2.7-fold range. Pairwise log-rank tests confirmed significantly slower resolution for structurally-driven symptoms: bruising vs itching *χ*^2^ (1) = 44.57, p = 2.5 × 10^−11^; bruising vs skin irritation *χ*^2^ (1) = 35.42, p = 2.7 × 10^−9^; and swelling vs walking difficulty *χ*^2^ (1) = 13.35, p < 0.001. Supplementary Figure S3 shows the 2 extreme survival curves with 95% CIs.

### Subdomain analysis

When grouped by pathophysiological subdomain, the persistence hierarchy was: vascular symptoms (bruising) and core inflammatory symptoms (swelling, heaviness, pressure) were most persistent, pain/sensory symptoms (touch sensitivity, pain) were intermediate, and skin symptoms (irritation, itching) and secondary vascular symptoms (cold, cramps) were most responsive. Notably, the 3 vascular subdomain symptoms (bruising, cold, cramps) showed divergent behavior: bruising was the most persistent of all 13 symptoms, while cold sensation and cramps were among the most responsive, suggesting that these symptoms reflect different aspects of vascular pathology.

## DISCUSSION

This retrospective observational study describes, for the first time, that all 13 inflammatory symptoms in lipedema improved during heterogeneous conservative management and followed a consistent resolution order across 6 complementary analyses in this cohort. The Friedman test (p < 0.001) confirmed that this ordering is statistically significant and consistent across patients. Six independent analytical approaches converged on a remarkably consistent ranking: cutaneous symptoms (irritation, itching) and walking difficulty respond most rapidly, while bruising and swelling improve more gradually but do improve meaningfully. A sensitivity analysis using the conventional first-to-last *T*_0_/*T*_1_ definition yielded a nearly identical ranking (Spearman ρ = 0.945), and a Kaplan-Meier analysis in absolute calendar time confirmed the same ordering (median 546 days for bruising vs 203 days for heat/burning to reach ≥ 50% reduction). These 3 independent analytical framings — magnitude-based, pair-dominance-based, and time-to-event-based — all converged on the same persistence hierarchy, supporting its internal validity.

### Bruising as the sentinel symptom

The finding that bruising is the most persistent symptom (ranking first in the consensus analysis, with a mean rank of 1.7) has both pathophysiological and clinical significance. In lipedema, easy bruising reflects capillary fragility, which Szolnoky et al. demonstrated can be objectively measured and used to differentiate lipedema from obesity.^23^ Studies have documented dilated and fragile blood vessels within lipedema tissue, with evidence of angiogenesis and increased vascular permeability.^10^ Al-Ghadban et al. described dilated blood microvessels and angiogenesis with macrophage infiltration in lipedema tissue,^10^ suggesting that the structural vascular changes underlying bruising may be more resistant to conservative treatment than the inflammatory mediators responsible for symptoms like heat/burning or skin irritation.

The persistence of bruising likely reflects the structural nature of the underlying pathology: while inflammatory mediators (responsible for heat, itching, and skin irritation) can be modulated relatively quickly, the microangiopathic changes responsible for capillary fragility may require longer treatment duration or more targeted interventions to resolve. This is consistent with the observation that bruising requires more time to reach 50% improvement than itching (normalized trajectory time 0.727 vs 0.525) — not because it fails to respond, but because structural vascular remodeling follows a slower time course than resolution of superficial cutaneous inflammation.

### Two distinct symptom tiers

Our analysis identified a clear dichotomy between a persistent core and a responsive periphery. The persistent core — bruising, swelling, touch sensitivity, leg heaviness, fatigue, pain, and pressure/tension — showed proportional reductions of 39%–49%, while the responsive periphery — cold, cramps, heat/burning, skin irritation, itching, and walking difficulty — showed reductions of 58%–66%. This approximately 1.4 to 1.7-fold difference in treatment responsiveness (group means: 62% vs 45%; extreme pair: 66.4% vs 39.1%) suggests fundamentally different pathophysiological mechanisms. Importantly, the bootstrap CI analysis confirmed that this 2-tier structure is statistically supported: the 95% CIs for rank 7 (pressure/tension: 4.33–7.00) and rank 8 (cold sensation: 8.00–9.17) did not overlap, representing the only non-overlapping boundary in the ranking. Within each tier, adjacent symptoms showed overlapping CIs, indicating that the tier membership is robust but the precise within-tier ordering should be interpreted cautiously.

The persistent symptoms appear to reflect structural tissue changes: bruising reflects capillary fragility, swelling reflects impaired lymphatic drainage and altered tissue sodium homeostasis,^2^ touch sensitivity and pain reflect peripheral nerve sensitization within the affected adipose tissue,^31^ leg heaviness and fatigue reflect the chronic burden of fluid accumulation and tissue inflammation, and pressure/tension reflects the mechanical consequence of interstitial fluid accumulation and connective tissue remodeling. These processes involve tissue remodeling that is slow to reverse.

In contrast, the responsive symptoms appear to reflect more labile inflammatory processes: heat/burning reflects acute vasodilation and local inflammatory mediator release, skin irritation and itching reflect superficial cutaneous inflammation, cold sensation and cramps reflect vasomotor instability, and walking difficulty reflects the functional consequence of acute symptom burden. These processes may respond more rapidly to anti-inflammatory interventions because they depend on circulating mediators rather than structural tissue changes.

Two symptoms showed notable dissociation between resolution persistence and re-emergence kinetics. Touch sensitivity (Q2) ranked third most persistent in the resolution hierarchy (consensus rank 4.3) but ninth in the emergence order (mean emergence time 0.745), suggesting that peripheral nerve sensitization, once suppressed by treatment, is slow to reactivate. Conversely, pressure/tension (Q4), despite being part of the persistent core (consensus rank 7 of 13), showed substantially greater treatment-responsiveness than bruising or swelling — with 48.9% proportional reduction vs 39–42% for the top 2 — yet it re-emerged as the second earliest symptom during worsening phases (emergence time 0.554, 72.7% of eligible patients). This dissociation suggests that pressure/tension reflects a highly reactive acute-phase inflammatory response: it is more amenable to intervention than structural vascular changes but quick to reactivate upon loss of inflammatory control.

To our knowledge, no prior study has characterized the order of symptom resolution in lipedema at an individual symptom level. Previous longitudinal studies — whether involving conservative treatment or liposuction^32^ — have reported composite outcomes (total symptom scores, quality-of-life scales, volume changes) without within-patient symptom-resolved kinetics. Our retrospective cohort adds this complementary perspective under conservative management.

Hierarchical symptom response is a recognized feature of chronic inflammatory diseases. In rheumatoid arthritis, clinical symptoms driven by active synovitis typically respond to disease-modifying therapy within weeks, while radiographic structural damage can progress even after clinical remission is attained — the canonical clinical-radiographic dissociation documented by Brown et al.^33^ In secondary lymphedema, the edematous component reduces substantially during the decongestive phase, whereas subcutaneous fibrosis is more treatment-resistant and typically requires prolonged maintenance interventions.^34^ Our lipedema findings — approximately 1.4-to-1.7-fold faster resolution for cutaneous and functional symptoms vs vascular-structural symptoms — fit this broader pattern of mediator-driven vs structurally-driven kinetics.

### Hypothesis-generating clinical implications

The following implications are hypothesis-consistent patterns from a retrospective cohort and should be regarded as exploratory, pending confirmation in prospective studies.

First, clinicians may consider informing patients that cutaneous symptoms (irritation, itching) and walking difficulty are likely to improve first under conservative treatment, whereas bruising and swelling may show more gradual change. Among strong responders in our cohort, 37.3% achieved complete or near-complete bruising resolution, while those with residual bruising still showed substantial overall improvement. This pattern raises the hypothesis that persistent bruising after weeks of conservative treatment should not be interpreted as treatment failure.

Second, the emergence order provides early warning markers for relapse. Our analysis showed that swelling and pressure/tension are the first symptoms to re-emerge during worsening phases, with 81.6% and 72.7% of patients experiencing their return, respectively. This hypothesis requires prospective evaluation before clinical adoption.

Third, the divergent behavior of vascular subdomain symptoms — bruising being the most persistent while cold and cramps are among the most responsive — suggests that these symptoms reflect different aspects of vascular pathology and should not be grouped together for treatment assessment purposes. Future revisions of the QuASiL subdomain taxonomy could consider separating capillary-fragility items from vasomotor items.

### Comparison with other chronic inflammatory conditions

While no prior study has examined symptom resolution order specifically in lipedema, the concept of hierarchical symptom response is recognized across chronic inflammatory conditions: symptoms driven by circulating mediators tend to respond faster than those reflecting structural tissue changes. Our findings in lipedema are consistent with this principle — symptoms driven by acute inflammation (heat/burning, skin irritation, itching) resolved approximately 1.4- to 1.7-fold faster than those reflecting structural pathology (capillary fragility, impaired lymphatic drainage, peripheral nerve sensitization) — and provide the first quantitative characterization of this hierarchy in a connective tissue disorder affecting adipose tissue.

### Strengths and limitations

This study has several strengths, including: (i) a large sample size (1,300 patients) drawn from a single specialist center, which ensured uniformity of clinical diagnosis; (ii) 6 independent analytical approaches yielding convergent results, which were reinforced by a new sensitivity analysis under the *T*_0_/*T*_1_ convention (Spearman *ρ* = 0.945 with primary ranking) and a Kaplan-Meier analysis in absolute calendar time; (iii) longitudinal data allowing assessment of both resolution and emergence patterns; and (iv) bootstrap confidence intervals around consensus ranks that explicitly quantify ranking uncertainty.

Five principal limitations constrain the interpretation of these findings: (i) its observational design and *T*_0_/*T*_1_ framing — the retrospective design precludes causal inference, and the primary worst-to-best *T*_0_/*T*_1_ convention biases the magnitude of change toward improvement; although the sensitivity analysis confirmed the ranking to be robust (Spearman ρ = 0.945), the absolute proportional reductions should be read as within-patient best response rather than average improvement; (ii) clinical characterization gaps — all patients received conservative treatment but specific regimens, adherence, and disease staging were not centrally recorded in the electronic database, so we cannot assess whether treatment modality or stage modifies the hierarchy; (iii) longitudinal structure — the ≥ 2-visit inclusion criterion is adequate to detect within-patient change but limited for characterizing temporal order; hence, analyses 3 and 4 and the Kaplan-Meier analysis were restricted to patients with ≥ 4 visits; the follow-up intervals varied widely (14 days to ~4.9 years; median ~6 months), reflecting real-world practice; (iv) patient-reported outcomes — although QuASiL scores are self-reported and subject to recall and response biases, they nevertheless remain the validated instrument for lipedema symptom assessment; (v) exploratory elements — the pathophysiological subdomain classification is investigator-proposed and the emergence analysis (N = 145) should be read as hypothesis-generating rather than definitive.

Future prospective studies with standardized treatment protocols, fixed assessment intervals, and documented disease staging are required to confirm whether the persistence hierarchy translates into prognostically useful guidance, and whether specific interventions preferentially target persistent vs responsive symptoms.

## CONCLUSIONS

In this retrospective cohort of 1,300 patients with a clinical diagnosis of lipedema under heterogeneous conservative management, the 13 QuASiL inflammatory symptoms improved following a consistent order across 6 complementary analyses, a sensitivity analysis under the conventional first-to-last *T*_0_/*T*_1_ definition (Spearman ρ = 0.945 with the primary ranking), and an absolute-time Kaplan-Meier survival analysis. Bruising and swelling emerged as the most persistent symptoms, whereas skin irritation, itching, walking difficulty, and heat/burning were the most responsive. The Kaplan-Meier analysis placed these differences in clinically interpretable units: a median of 546 days for bruising vs 203 days for heat/burning to reach a ≥ 50% reduction from baseline, with log-rank p < 10^−10^ at the extreme pair.

Given the observational design, the lack of a standardized treatment protocol or systematic disease staging, and the heterogeneity of follow-up intervals, these findings should be interpreted as exploratory and hypothesis-generating. The suggestion that cutaneous and functional symptoms may serve as early treatment-response indicators and that swelling or pressure/tension may serve as early relapse markers requires confirmation in prospective studies with standardized interventions, fixed assessment schedules, and documented staging before clinical adoption.

## Data Availability

Clinical data are not made available due to ethical restrictions regarding patient privacy. The analysis code is available upon request to the corresponding author.

## Supplementary Material

Supplementary material accompanies this paper.

Table S1 Follow-up interval (days) by analysis subgroup.

Table S2 Sensitivity analysis — consensus persistence ranking in which *T*_0_ = first visit and *T*_1_ = last visit. Analysis population: improvers (*Δ* inflammatory > 10 between first and last visit), N = 566.

Table S3 Rank concordance — primary (worst-best) vs sensitivity (first-last) consensus ranking.

Table S4 Kaplan-Meier median time (days) to clinical events by symptom. Analysis population: patients with ≥ 4 QuASiL assessments and baseline symptom scores ≥ 3. Event A = first visit at which the score dropped to ≤ 50% of baseline. Event B = first visit at which the score dropped to ≤ 2 (mild/absent). Patients not reaching the threshold were censored at their last assessment.

FIGURE S1 Sensitivity analysis: comparison of primary (worst-to-best) vs conventional (first-to-last) persistence rankings. Each point represents 1 symptom; dashed line = perfect concordance; Spearman ρ = 0.945.

FIGURE S2 Kaplan-Meier survival curves for all 13 QuASiL inflammatory symptoms (time to ≥ 50% reduction from baseline). Bruising (upper curve) and swelling had the slowest resolution, while heat/burning and cold sensation had the fastest. Log-rank test: bruising vs itching *χ*^2^ = 44.57, p < 10^−10^.

FIGURE S3 Kaplan-Meier curves for the 2 extreme symptoms (bruising vs itching) with 95% CIs. Median time to ≥ 50% reduction: bruising 546 days, itching 207 days.

This material is available as part of the online article from https://doi.org/10.1590/1677-5449.202600562
