# Supplementary material for: Order of resolution and recurrence of inflammatory symptoms in lipedema: a longitudinal study of 1,300 patients
Source: J Vasc Bras. 2026 Sep 7;25:e20260056. doi: 10.1590/1677-5449.202600562 (PMC13671008; doi:10.1590/1677-5449.202600562)

FIGURE S1. Sensitivity analysis: comparison of primary (worst-to-best) vs conventional (first-to-last) persistence rankings. Each point represents 1 symptom; dashed line = perfect concordance; Spearman ρ = 0.945.

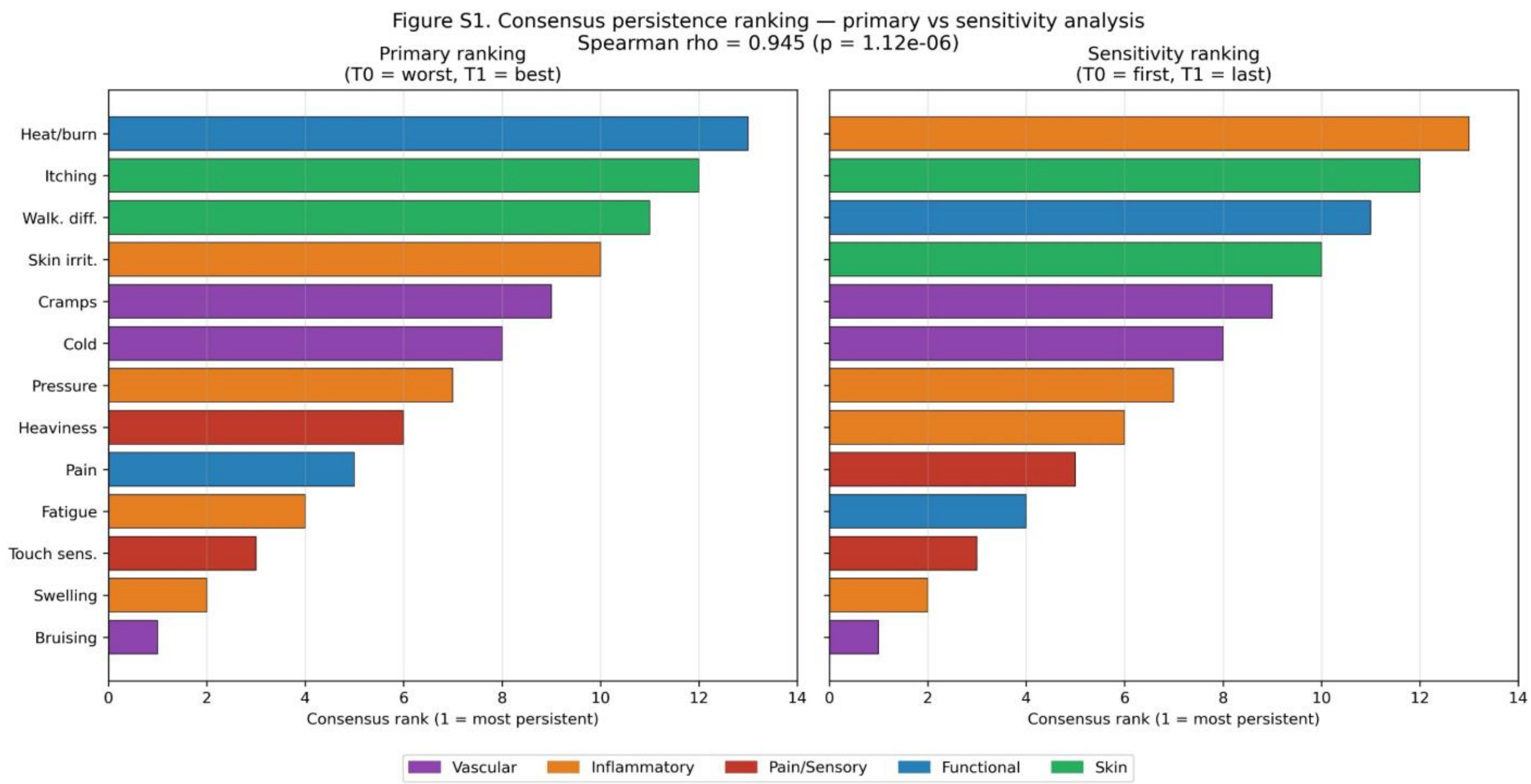

Supplement: Supplementary file 1 [file jvb-25-e20260056-suppl001.pdf]
