# Supplementary material for: Order of resolution and recurrence of inflammatory symptoms in lipedema: a longitudinal study of 1,300 patients
Source: J Vasc Bras. 2026 Sep 7;25:e20260056. doi: 10.1590/1677-5449.202600562 (PMC13671008; doi:10.1590/1677-5449.202600562)

FIGURE S2. Kaplan-Meier survival curves for all 13 QuASiL inflammatory symptoms (time to ≥ 50% reduction from baseline). Bruising (upper curve) and swelling had the slowest resolution, while heat/burning and cold sensation had the fastest. Log-rank test: bruising vs itching $\chi^2 = 44.57$, $p < 10^{-10}$.

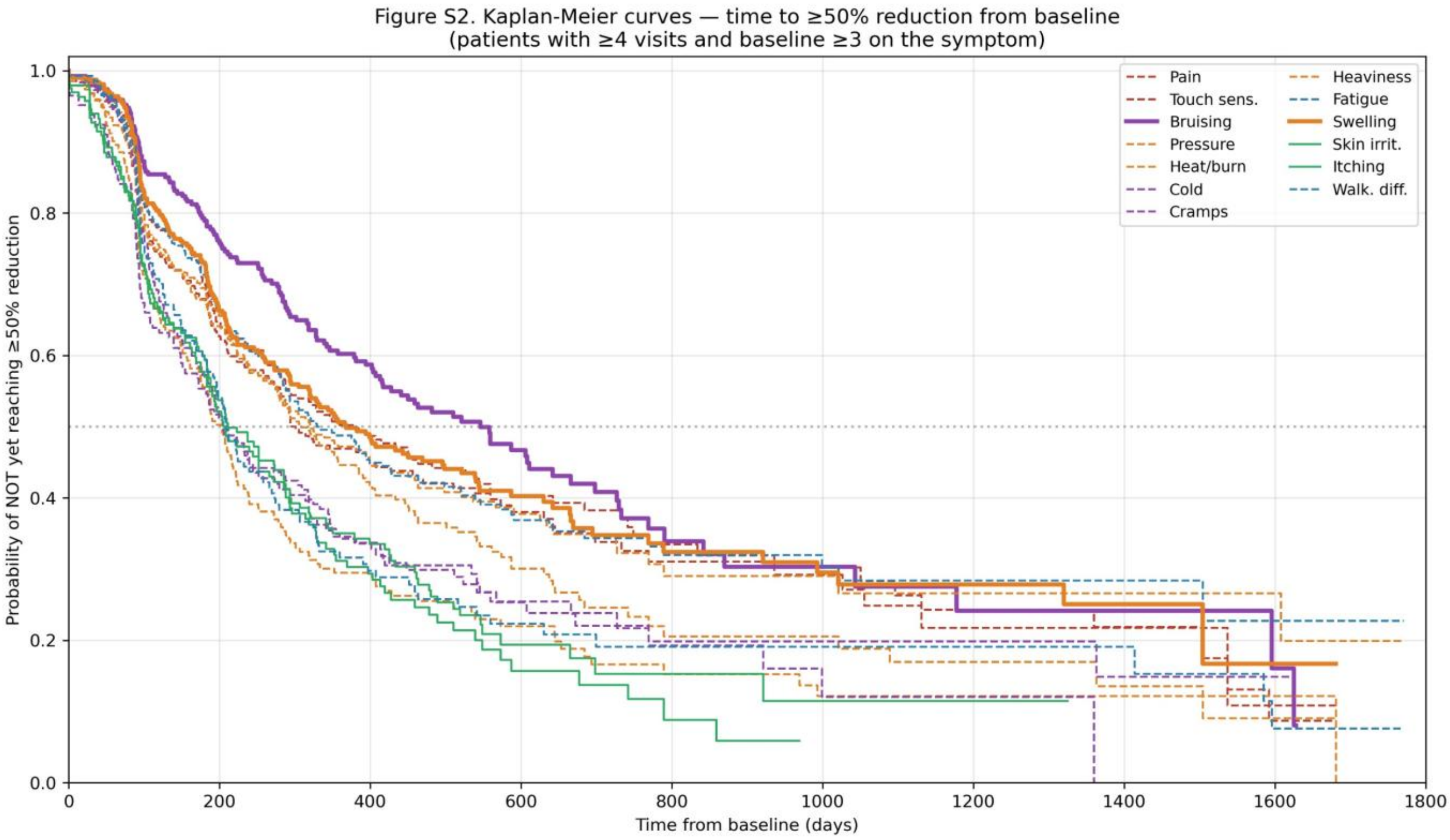

Supplement: Supplementary file 2 [file jvb-25-e20260056-suppl002.pdf]
