# Supplementary material for: Order of resolution and recurrence of inflammatory symptoms in lipedema: a longitudinal study of 1,300 patients
Source: J Vasc Bras. 2026 Sep 7;25:e20260056. doi: 10.1590/1677-5449.202600562 (PMC13671008; doi:10.1590/1677-5449.202600562)

FIGURE S3. Kaplan-Meier curves for the 2 extreme symptoms (bruising vs itching) with 95% CIs. Median time to ≥ 50% reduction: bruising 546 days, itching 207 days.

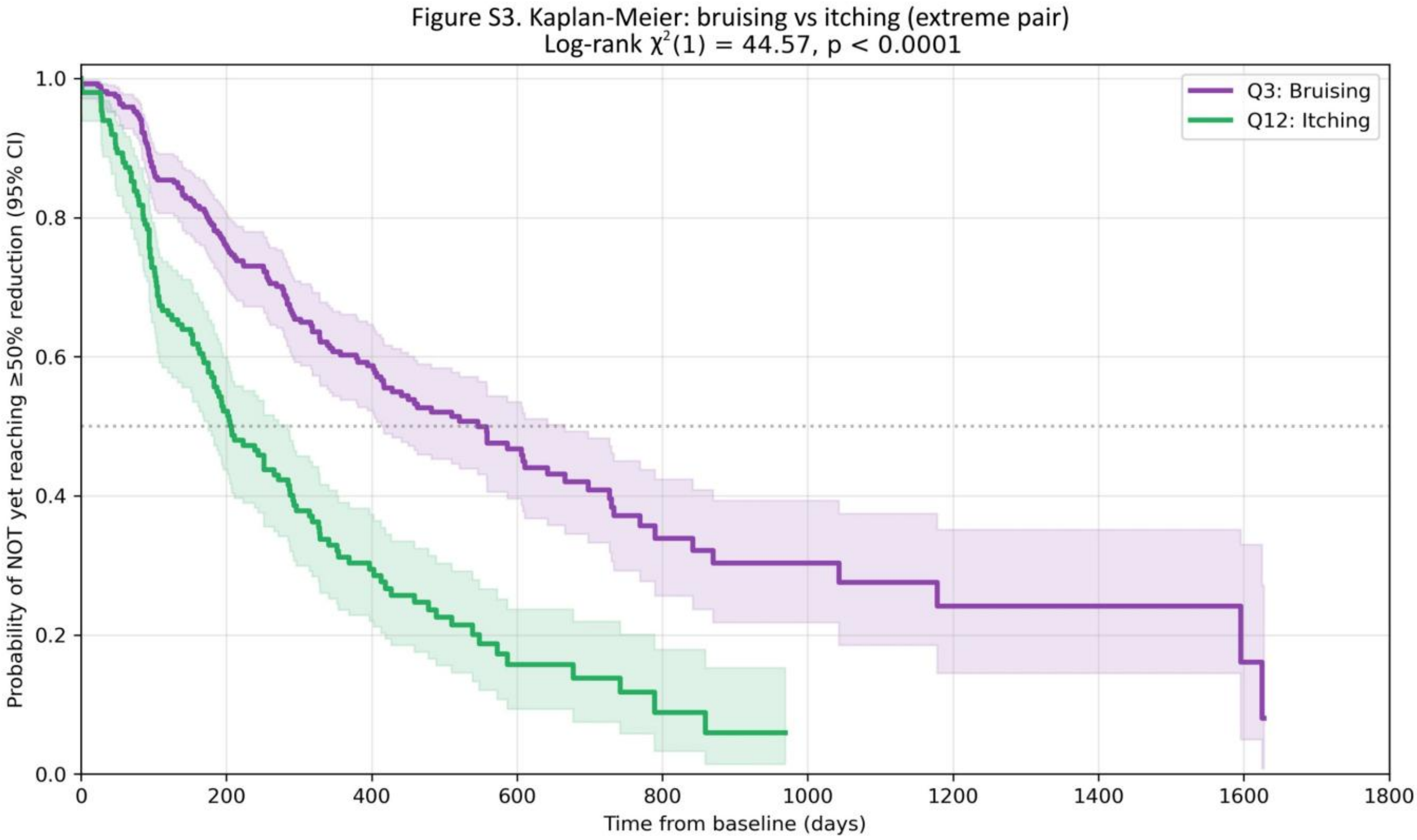

Supplement: Supplementary file 3 [file jvb-25-e20260056-suppl003.pdf]
