# Supplementary material for: Order of resolution and recurrence of inflammatory symptoms in lipedema: a longitudinal study of 1,300 patients
Source: J Vasc Bras. 2026 Sep 7;25:e20260056. doi: 10.1590/1677-5449.202600562 (PMC13671008; doi:10.1590/1677-5449.202600562)
Supplement: Supplementary file 4 [file jvb-25-e20260056-suppl01.pdf]

Table S1. Follow-up interval (days) by analysis subgroup.

Analysis subgroup	N	Min.	Q1	Median	Q3	Max.	IQR	Mean (SD)
Entire longitudinal cohort	1,300	14	92	181	380	1,787	288	276 (295)
Improvers (Δ inflammatory > 10)	660	14	105	250	467	1,787	362	341 (328)
Strong responders (top 25%, $\Delta \geq 30$)	335	25	150	304	556	1,787	406	391 (336)
≥ 4 visits (trajectory eligible)	331	52	315	489	744	1,787	429	533 (305)
≥ 4 visits AND improved	196	82	317	481	772	1,787	455	548 (311)
Worsening-phase subset (Analysis 4)	145	52	446	650	993	1,787	547	743 (375)

All subgroups exclude patients with intervals < 14 days (primary inclusion filter).
