# Supplementary material for: Order of resolution and recurrence of inflammatory symptoms in lipedema: a longitudinal study of 1,300 patients
Source: J Vasc Bras. 2026 Sep 7;25:e20260056. doi: 10.1590/1677-5449.202600562 (PMC13671008; doi:10.1590/1677-5449.202600562)
Supplement: Supplementary file 5 [file jvb-25-e20260056-suppl02.pdf]

Table S2. Sensitivity analysis — consensus persistence ranking in which T_0 = first visit and T_1 = last visit. **Analysis population:** improvers (Δ inflammatory > 10 between first and last visit), N = 566.

**Rank**	**Symptom (QuASiL item)**	**Subdomain**	**Mean rank (across 5 analyses)**	**SD**
1	Q3: Bruising	Vascular	1.4	0.49
2	Q10: Swelling	Inflammatory	2.2	1.17
3	Q2: Touch sensitivity	Pain/Sensory	3.4	1.36
4	Q9: Fatigue	Functional	4.2	0.98
5	Q1: Pain	Pain/Sensory	5.0	1.67
6	Q8: Leg heaviness	Inflammatory	5.4	1.20
7	Q4: Pressure/tension	Inflammatory	6.4	0.49
8	Q6: Cold sensation	Vascular	8.0	0.00
9	Q7: Cramps	Vascular	9.8	1.17
10	Q11: Skin irritation	Skin	10.6	1.20
11	Q13: Walking difficulty	Functional	11.2	1.17
12	Q12: Itching	Skin	11.4	0.80
13	Q5: Heat/burning	Inflammatory	12.0	1.55

Friedman test for the sensitivity cohort (patients with all 13 symptoms active at first visit, N = 80): χ^2 (12) = 63.65, p = 4.9 × 10^{-9}. Spearman rank correlation with the primary consensus ranking (Table 6): ρ = 0.945, p = 1.1 × 10^{-6}. The emergence analysis (Analysis 4), which used the raw multi-visit trajectory, remained unchanged and has thus not been repeated here.
