# Supplementary material for: Order of resolution and recurrence of inflammatory symptoms in lipedema: a longitudinal study of 1,300 patients
Source: J Vasc Bras. 2026 Sep 7;25:e20260056. doi: 10.1590/1677-5449.202600562 (PMC13671008; doi:10.1590/1677-5449.202600562)
Supplement: Supplementary file 6 [file jvb-25-e20260056-suppl03.pdf]

Table S3. Rank concordance — primary (worst-best) vs sensitivity (first-last) consensus ranking.

**Symptom**	**Primary rank**	**Sensitivity rank**	**Δ**
Q3: Bruising	1	1	+0
Q10: Swelling	2	2	+0
Q2: Touch sensitivity	3	3	+0
Q8: Leg heaviness	4	6	+2
Q9: Fatigue	5	4	−1
Q1: Pain	6	5	−1
Q4: Pressure/tension	7	7	+0
Q6: Cold sensation	8	8	+0
Q7: Cramps	9	9	+0
Q5: Heat/burning	10	13	+3
Q11: Skin irritation	11	10	−1
Q12: Itching	12	12	+0
Q13: Walking difficulty	13	11	−2

Spearman $\rho = 0.945$, $p = 1.1 \times 10^{-6}$. Max |rank shift| = 3 positions.
