# Supplementary material for: Order of resolution and recurrence of inflammatory symptoms in lipedema: a longitudinal study of 1,300 patients
Source: J Vasc Bras. 2026 Sep 7;25:e20260056. doi: 10.1590/1677-5449.202600562 (PMC13671008; doi:10.1590/1677-5449.202600562)
Supplement: Supplementary file 7 [file jvb-25-e20260056-suppl04.pdf]

Table S4. Kaplan-Meier median time (days) to clinical events by symptom. **Analysis population:** patients with ≥ 4 QuASiL assessments and baseline symptom scores ≥ 3. Event A = first visit at which the score dropped to ≤ 50% of baseline. Event B = first visit at which the score dropped to ≤ 2 (mild/absent). Patients not reaching the threshold were censored at their last assessment.

**Rank**	**Symptom (QuASiL item)**	**Subdomain**	**N**	**Events (≥ 50%)**	**Median days to ≤ 50% baseline**	**Events (≤ 2)**	**Median days to ≤ 2**
1	Q3: Bruising	Vascular	270	139	546	93	870
2	Q2: Touch sensitivity	Pain/Sensory	272	158	381	125	684
3	Q10: Swelling	Inflammatory	309	177	368	94	1,608
4	Q9: Fatigue	Functional	306	179	332	120	924
5	Q8: Leg heaviness	Inflammatory	291	172	321	117	789
6	Q4: Pressure/tension	Inflammatory	276	184	306	126	637
7	Q1: Pain	Pain/Sensory	278	165	301	120	769
8	Q7: Cramps	Vascular	187	132	211	129	237
9	Q11: Skin irritation	Skin	165	123	211	111	265
10	Q13: Walking difficulty	Functional	143	107	209	98	266

Rank	Symptom (QuASiL item)	Subdomain	N	Events (≥ 50%)	Median days to ≤ 50% baseline	Events (≤ 2)	Median days to ≤ 2
11	Q12: Itching	Skin	149	116	207	98	287
12	Q6: Cold sensation	Vascular	145	104	205	88	294
13	Q5: Heat/burning	Inflammatory	193	152	203	135	232

Ranked by median days until reaching ≤ 50% of baseline (descending; rank 1 = slowest = most persistent).

Log-rank tests on selected pairs (event = reach ≤ 50% baseline):

Pair	χ^2 (1)	p
Q3: Bruising vs Q12: Itching	$44.^{57}$	$2.^{5} \times 10^{-11}$
Q3: Bruising vs Q11: Skin irritation	$35.^{42}$	$2.^{7} \times 10^{-9}$
Q10: Swelling vs Q11: Skin irritation	$18.^{80}$	$1.^{5} \times 10^{-5}$
Q10: Swelling vs Q13: Walking difficulty	$13.^{35}$	$2.^{6} \times 10^{-4}$
Q3: Bruising vs Q13: Walking difficulty	$26.^{66}$	$2.^{4} \times 10^{-7}$
